# Protocol for measuring the caspase-8 activity at the DED filaments in adherent cells

**DOI:** 10.1016/j.xpro.2025.104131

**Published:** 2025-10-08

**Authors:** Corinna König, Inna N. Lavrik

**Affiliations:** 1 Translational Inflammation Research, Medical Faculty, Center of Dynamic Systems, Otto von Guericke University, Magdeburg, Germany

**Keywords:** Cell Biology, Cell-based Assays, Cancer

## Abstract

The key stage of extrinsic apoptosis is the activation of procaspase-8 at the death-inducing signaling complex (DISC), where procaspase-8 assembles into death effector domain (DED) filaments. Here, we present a protocol to measure caspase-8 activity directly at the DISC. We describe steps for cell culture, apoptosis induction, immunoprecipitation, caspase-8 assay, and western blot analysis. This approach enables the analysis of caspase-8 activation in its native complex and can be applied to assess the efficacy of pharmacological inhibitors targeting caspase-8.

For complete details on the use and execution of this protocol, please refer to König et al.^1^

## Highlights

• Steps for measuring caspase-8 activity at the death-inducing signaling complex (DISC)
• Instructions for combining immunoprecipitation (IP) with a caspase-8 activity assay
• Analysis of IP efficiency and caspase-8 enzymatic activity from the same sample

## Abstract (editor highlights)

Publisher’s note: Undertaking any experimental protocol requires adherence to local institutional guidelines for laboratory safety and ethics.

## Graphical abstract

**Figure:**
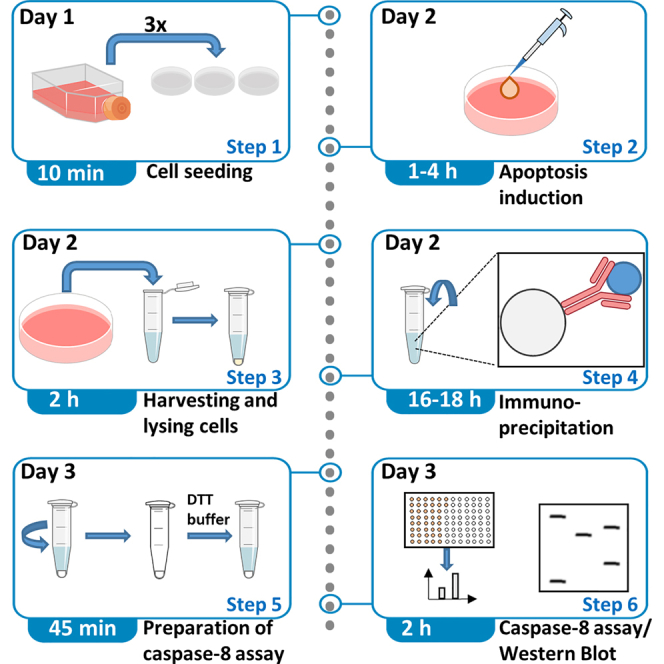

## Before you begin

### Innovation

The programmed cell death is an important process to maintain the homeostasis in multicellular organisms. Extrinsic apoptosis, a form of programmed cell death, is initiated by the binding of a death ligand (DL) to a corresponding death receptor (DR).^2^^,^^3^^,^^4^^,^^5^ CD95/Fas is a DR belonging to the TNFR superfamily.^5^^,^^6^^,^^7^ After the binding of CD95L to CD95 the adaptor protein FADD is recruited to the death domain (DD) of DR with its own DD.^3^^,^^8^^,^^9^^,^^10^ FADD also contains a death effector domain (DED), which allows to recruit other DED proteins like procaspase-8.^11^ The complex of DR, FADD and DED proteins is called the death-inducing signaling complex (DISC), which provides the platform for procaspase-8 activation.^11^^,^^12^ Procaspase-8 forms DED filaments at the DISC which is crucial for its activation at the DISC.^11^^,^^12^^,^^13^^,^^14^^,^^15^ Caspase-8 activation is one of the key events in induction of extrinsic apoptosis.

To analyze the induction of extrinsic apoptosis it is necessary to evaluate the caspase-8 activation at the DISC.^14^^,^^16^^,^^17^ Recently we reported a pioneering probe, rationally designed peptide, DEDid, that binds to procaspase-8 DED, which leads to blocking the DED filament formation.^1^ These results were supported by generation of procaspase-8 mutants at DEDid binding site of procaspase-8. This protocol provides the opportunity to analyze how various ways to target caspase-8 alter the caspase-8 activation upon CD95L treatment at the DISC.^1^^,^^18^^,^^19^^,^^20^ From this data, we could conclude how interfering with the DED filament assembly influences caspase-8 activity.

Here we describe the protocol using HeLa-CD95 (CD95-overexpression) cells as the exemplary model.^21^ The protocol also can be used for measuring caspase-8 activation in other adherent cells lines as well as for suspension cell lines.

Clearly label and date all consumables and reagents.

Choose appropriate cell lines for the analysis. Selected candidates should be sensitive to CD95L-induced apoptosis, which should be tested prior to experiments.

Ensure that all cell cultures to be used are free from contamination, including mycoplasma and other microorganisms.

### Preparation of cell culture

**Timing: 2 days**

1. Addition of supplements to the DMEM F12 medium.
  a. Add Penicillin/Streptomycin to the medium to a final concentration of 0.1 mg/mL.
  b. Add puromycin to a final concentration of 0.2 μg/mL.
  c. Add fetal calf serum (FCS) to the medium to a final amount of 10%.

***Note:*** Work under sterile conditions.

***Note:*** Supplement concentrations may vary depending on the cell type used.

2. Pre-warm the DMEM F12 medium with supplements.
  a. Pre-warm the medium to 37°C before adding it to the cells.
3. Seed cells in a suitable cell culture flask.
  a. Seed 2 x 10^6^ HeLa-CD95 cells in 30 mL medium in a T175 flask.
  b. Incubate cells for two days at 37°C with 5% CO_2._

***Note:*** We recommend using T175 flasks for either adherent or suspension cells, depending on the specific cell line. Seed an appropriate number of cells to ensure sufficient yield for all experimental conditions.

***Note:*** Cell seeding numbers may vary depending on the specific cell line used.

### Cell culture and preparation for seeding

**Timing: 1 day**

4. Remove medium from the cell culture flask.
  a. Add 10 mL of PBS to rinse the cells.
  b. Immediately aspirate and discard the PBS.
  c. Add 2 mL of trypsin to the flask to detach the cells.
    i. Incubate for 5 min in the cell culture incubator at 37°C with 5% CO_2._

***Note:*** The trypsinization step may take up to 10 min, depending on the cell type.

***Note:*** Trypsinization is not needed when suspension cells are used. Suspension cells can be counted directly.

5. Add 10 mL of medium to the flask to neutralize the trypsin after trypsinization.
  a. Transfer cell suspension to a 50 mL centrifuge tube.
  b. Centrifuge cells at 500 × *g* for 5 min at room temperature (RT).

***Note:*** Centrifugation speed and duration may be adjusted based on the specific cell type used.

6. Carefully discard the supernatant without disturbing the cell pellet.
  a. Add 10 mL of fresh medium to the cell pellet.
  b. Gently resuspend the cell pellet in the medium by pipetting up and down until fully dispersed.
  c. Mix the cells with Trypan Blue at a 1:2 ratio to stain them.
  d. Count the stained cells using a suitable commercial cell counter or a Neubauer chamber and distinguish between viable and dead cells.

**CRITICAL:** In this step, check the cell viability and proceed only with samples that have a viability greater than 93%.

7. Seed 5 x 10^6^ HeLa-CD95 cells in 14.5 cm plates with 20 mL medium.
  a. Three plates per one condition (triplicates).
    i. One for Western Blot control, two for duplicates for caspase-8 assay.
    ii. For ‘Beads control’ only one plate is necessary.
  b. Let the plates with the cells stay overnight in the cell culture incubator at 37°C with 5% CO_2_.
  c. The number of cells used my be adjusted – more or fewer - depending on the cell line.
  d. For example we would recommend for HT29 cells 8 x 10^6^ cells per plate.
  e. For suspension cells we recommend 1 x 10^7^ cells per condition, seeded on the day of the stimulation.

***Note:*** Suspension cells do not require overnight incubation and can be used immediately for treatment.

***Note:*** The cells are prepared for ‘Beads control,’ which serves as a control for nonspecific binding in immunoprecipitation.

## Key resources table

**Table:** 

REAGENT or RESOURCE	SOURCE	IDENTIFIER
**Antibodies**		

Rabbit polyclonal anti-caspase-3, diluted 1:2,000	Cell Signaling Technology	#9662, RRID: AB_331439
Rabbit polyclonal anti-PARP1, diluted 1:1,000	Cell Signaling Technology	#9542, RRID: AB_2160739
Rabbit monoclonal anti-RIPK1 XP, diluted 1:1,000	Cell Signaling Technology	#3493, RRID: AB_2305314
Rabbit polyclonal anti-actin, diluted 1:4,000	Sigma-Aldrich	A2103, RRID: AB_476694
Mouse monoclonal anti-CD95, diluted 1:500	Santa Cruz	sc-8009, RRID: AB_627218
Mouse monoclonal anti-caspase-10, diluted 1:1,000	MBL International Corporation	M059-3, RRID: AB_590721
Mouse monoclonal anti-FADD (clone 1C4), diluted 1:10	Muzi et al.^22^	N/A
Mouse monoclonal anti-caspase-8 (clone C15), diluted 1:20	Scaffidi et al.^23^	N/A
Mouse monoclonal c-FLIP (clone NF6), diluted 1:10	Scaffidi et al.^23^	N/A
Rabbit monoclonal anti-APO-1 antibody (mouse-IgG3)	Muzio et al.^22^	N/A
Horseradish peroxidase-conjugated goat anti-mouse IgG1	SouthernBiotech	1070-05, RRID: AB_2650509
Horseradish peroxidase-conjugated goat anti-mouse IgG2b	SouthernBiotech	1090-05, RRID: AB_2794521
Horseradish peroxidase-conjugated goat anti-rabbit	SouthernBiotech	4030-05, RRID: AB_2687483

**Chemicals, peptides, and recombinant proteins**		

CD95L	Fricker et al.^24^	N/A
DTT	OmniLab	APP A2948
CHAPS	OmniLab	APP A1099
Mercaptoethanol	Carl Roth	4227.2
HEPES	OmniLab	APP A3268
NaCl	Carl Roth	3957.2
KCl	Carl Roth	6781.2
Na_2_HPO_4_	Carl Roth	P030.3
KH_2_PO_4_	Carl Roth	3904.1
Tween 20	PanReac AppliChem	A4974
Milk powder	Carl Roth	T145.4
EDTA	Carl Roth	3054.1
Triton X-100	Carl Roth	3051.4
Tris	Chem Solute	8085.1000
Glycin	Carl Roth	3908.3
Glycerol	Carl Roth	3783.1
HCl	Carl Roth	4326.1
SDS	Carl Roth	4360.2
Acrylamide/Bisacrylamide (29:1)	Carl Roth	A124.1
TEMED	Carl Roth	2367.3
APS	Carl Roth	9592
Isopropanol	Carl Roth	6752.5
Protease Inhibitor Cocktail	Roche	11836145001
BSA	PanReac AppliChem	A6588
NaN_3_	Carl Roth	K305.1
Protein A Sepharose Beads	Cytiva	17-0780-01
Lämmli sample buffer	Bio-Rad	161-0747
Precision Plus Protein Standards All Blue	Bio-Rad	161-0373
Bradford Protein Assay	Bio-Rad	500-0006
Luminata Forte Western HRP substrate	Millipore	WBLUFO500

**Critical commercial assays**		

Caspase-Glo 8 Assay	Promega	G8201
Transblot Turbo Mini Size Kit	Bio-Rad	170-4270

**Experimental models: Cell lines**		

Human: HeLa-CD95	Neuman et al.^21^	N/A
DMEM Hams F12	Pan Biotech	P04-41150
FCS	Thermo Fisher Scientific, Inc.	10270-106
Penicillin Streptomycin	Biochrom	A2213
Puromycin	Invitrogen	Ant-pr-1
PBS	Pan Biotech	P04-36500
Trypsin	Thermo Fisher Scientific, Inc.	25300-096
Trypan blue	Thermo Fisher Scientific, Inc.	15250-061

**Software and algorithms**		

Graphpad Prism 8	GraphPad	https://www.graphpad.com/
Image Lab	Bio-Rad	https://www.bio-rad.com/de-de/product/image-lab-software?ID=KRE6P5E8Z

**Other**		

Photometer GeneQuant 1300	GE	https://www.gehealthcare.de/
TECAN infinite M200 PRO	Tecan	https://www.tecan.de/
Chemiluminescence Imager	Bio-Rad	https://www.bio-rad.com/
PowerPac HC	Bio-Rad	https://www.bio-rad.com/
Trans-Blot-Turbo	Bio-Rad	https://www.bio-rad.com/

## Materials and equipment

**Table:** Lysis buffer

Reagent	Amount	Final concentration
1.5 M Tris HCl, pH 7.4	13.3 mL	19.95 mM
5 M NaCl	27.5 mL	137.5 mM
0.2 M EDTA, pH 8.0	10 mL	2 mM
1.71 M Triton X-100	100 mL	17.1 mM
Glycerol	100 mL	1.37 M (10% v/v)
H_2_O	Ad 1000 mL	N/A
**Total**	1000 mL	N/A
Protease Inhibitor Cocktail (PIC)	Add 4% fresh to 96% lysis buffer	N/A

***Note:*** Store it at 4°C up to 1 year.

***Note:*** For cell lysis use a mixture of 96% lysis buffer and 4% PIC.

**Table:** CHAPS solution

Reagent	Amount	Final concentration
CHAPS	10 mg	1 mg/mL (∼1.63 mM)
H_2_O	Ad 10 mL	N/A
**Total**	10 mL	N/A

***Note:*** Make it always fresh.

**Table:** HEPES buffer

Reagent	Amount	Final concentration
1 M HEPES, pH 7.2	2.5 mL	50 mM
5 M NaCl	0.5 mL	50 mM
0.2 M EDTA, pH 8.0	2.5 mL	10 mM
Glycerol	2.5 mL	685 mM (5% v/v)
H_2_O	Ad 50 mL	N/A
**Total**	50 mL	N/A

***Note:*** Store it at 4°C up to 1 month.

**Table:** DTT buffer, fresh

Reagent	Amount	Final concentration
DTT	0.0031 g	10 mM
1 M HEPES buffer, pH 7.2	2 mL	1 M
CHAPS solution	2 μL	1.63 μM (0.1% v/v)
**Total**	2 mL	N/A

***Note:*** Make it always fresh.

**Table:** 10× electrophoresis buffer

Reagent	Amount	Final concentration
Tris	60.6 g	0.25 M
Glycin	288 g	1.92 M
SDS	20 g	34.7 mM
H_2_O	Ad 2000 mL	N/A
**Total**	2000 mL	N/A

***Note:*** Store it at RT for up to 1 month.

***Note:*** Dilute the 10× buffer to 1× buffer with H_2_O.

**Table:** 12.5% separating gel

Reagent	Amount	Final concentration
H_2_O	1.64 mL	N/A
1.5 M Tris, pH 8.8	1.25 mL	375 mM
Acrylamide/Bisacrylamide	2.03 mL	40.6% v/v
350 mM SDS (10%)	50 μL	3.5 mM (1% v/v)
868 mM APS (10%)	50 μL	8.68 mM (1% v/v)
1.67 M TEMED	3.75 μL	1.25 mM
**Total**	5 mL	N/A

***Note:*** Add everything in the order as it appears in the table.

***Note:*** Add 1 mL of isopropanol on top of the gel immediately after pouring it between the glass plates, before it begins to polymerize. This helps to create a smooth, even surface and prevent bubble formation.

***Note:*** The table shows the amounts of reagents for one gel.

**CRITICAL:** Acrylamide/Bisacrylamide and TEMED are toxic and hazardous to human health. Always wear gloves when handling these reagents and dispose of the gloves immediately after exposure. TEMED should be handled under proper ventilation or an air extraction system. Note that the polyacrylamide gel becomes significantly less toxic once fully polymerized.

**Table:** Stacking gel

Reagent	Amount	Final concentration
H_2_O	1.55 mL	N/A
1.5 M Tris, pH 6.8	0.625 mL	375 mM
Acrylamide/Bisacrylamide	0.25 mL	10% v/v
350 mM SDS (10%)	25 μL	3.5 mM (1% v/v)
868 mM APS	12.5 μL	4.34 mM (1% v/v)
1.67 M TEMED	3.75 μL	2.5 mM
**Total**	2.5 mL	N/A

***Note:*** Store at 4°C for 1 day, store the gel in 10 mL 1× Electrophoresis buffer.

***Note:*** Add everything in the order as it appears in the table.

***Note:*** Insert an appropriate comb to form sample wells immediately after pouring the gel solution between the glass plates, before it begins to solidify.

***Note:*** The table shows the amounts of reagents for one gel.

**CRITICAL:** Acrylamide/Bisacrylamide and TEMED are toxic and hazardous to human health. Always wear gloves when handling these reagents and dispose of the gloves immediately after exposure. TEMED should be handled under proper ventilation or an air extraction system. Note that the polyacrylamide gel becomes significantly less toxic once fully polymerized.

**Table:** 20× PBST

Reagent	Amount	Final concentration
NaCl	320 g	2,74 M
KCl	8 g	54 mM
Na_2_HPO_4_	56.8 g	200 mM
KH_2_PO_4_	8 g	29 mM
Tween-20, pH 7	20 mL	89 mM (10% v/v)
H_2_O	Ad 2000 mL	N/A
**Total**	2000 mL	N/A

***Note:*** Store it at RT for up to 1 month.

***Note:*** Dilute the 20× buffer to 1× buffer with H_2_O.

**Table:** 5% milk in PBST

Reagent	Amount	Final concentration
Milk powder	50 g	50 mg/mL (5% w/v)
PBST	Ad 1000 mL	N/A
**Total**	1000 mL	N/A

***Note:*** Store it at 4°C for up to 1 week.

## Step-by-step method details

### Treating the cells for apoptosis induction

**Timing: 2–4 h (depending on the time of the treatment)**

This step involves treating the cells according to the experimental design, which should specify the compounds, concentrations, and time points relevant to the study.

1. Remove medium from the 14.5 cm plates.
  a. Add 10 mL of fresh medium to the 14.5 cm plates.
  b. Add the required concentration of the compound you want to use.
    i. We used 125 ng/mL CD95L.
  c. Incubate the cells in the cell culture incubator at 37°C with 5% CO_2_ for the required time intervals.
    i. We incubated the cells for 2 h.

**Figure 1:**
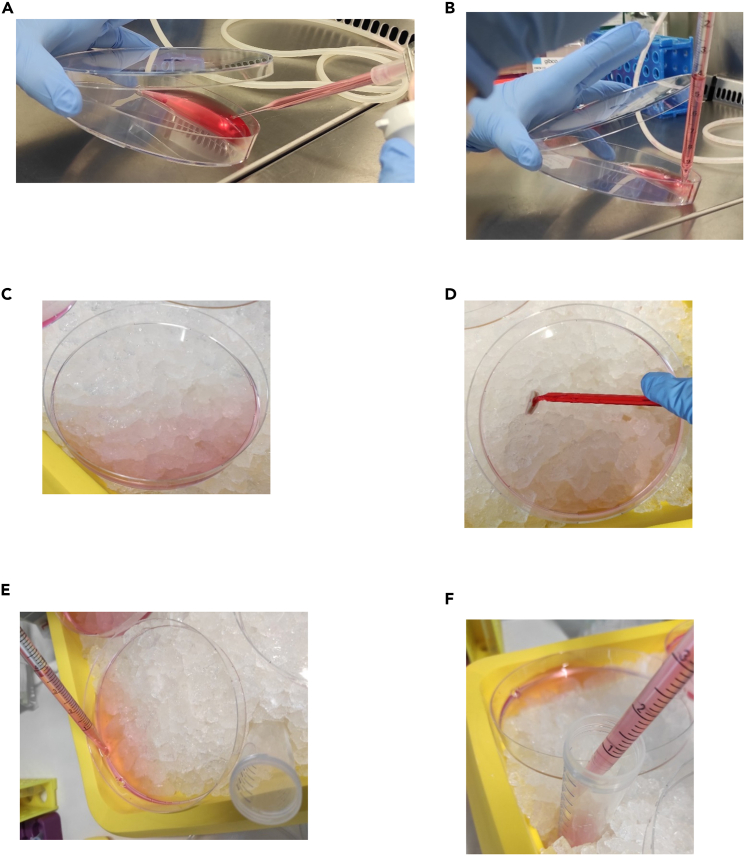
How to treat and harvest the cells (A) Picture of how to discard the media from adherent cells without stressing the cells or detach the cells from the plate. The tip of the aspiration pipette is in the edge of the plate. Lid is only slightly opened to ensure sterile conditions. (B) Picture of how to add fresh medium to adherent cells without stressing the cells or detach the cells from the plate. The tip of the pipette is in the edge of the plate. Lid is only slightly opened to ensure sterile conditions. (C) Before harvesting the cells place them on ice. (D) Scrap all the adherent cells from the plate with a cell scraper. Work on ice. (E and F) Transfer the cell suspension into a 50 mL tube. Work on ice.

**CRITICAL:** Depending on the cell line used, some adherent cells may be sensitive and can detach during medium changes. To minimize cell loss, carefully discard the medium and add fresh medium by pipetting in the edge of the well or plate (see Figures 1A and 1B).

***Note:*** Changing the medium is only necessary for adherent cells. Suspension cells can be treated directly in the medium in which they were seeded.

***Note:*** Preliminary experiments may be necessary to determine the optimal time points and concentrations for specific stimulation agents.

***Note:*** Be sure to treat the ‘Beads control’ under the same conditions.

### Harvesting the cells and cell lysis

**Timing: 2 h**

In this step, the cells are harvested, lysed and prepared for the Immunoprecipitation (IP). Start with this step after accomplishing the required time of stimulation.

2. Place the 14.5 cm plates on ice after stimulation (in our case 2 h) (Figure 1C).
  a. Add 10 mL of ice cold PBS to every plate.
  b. Scrap the cells from the surface with a cell scraper (Figure 1D).
    i. Scrap the whole surface, also in the edges of the plate.
  c. Transfer the cell suspension in a 50 mL tube (Figures 1E and 1F).
  d. Wash the plate two times with 10 mL PBS.
    i. Transfer the PBS/cell suspension in the corresponding 50 mL tube.

***Note:*** Work the whole time on ice (Figure 1C).

***Note:*** Suspension cells do not need to be scraped.

**CRITICAL:** Do not discard the medium before adding PBS, otherwise you risk losing cells.

**CRITICAL:** Do not merge the same conditions, treat them as separate samples.

**Figure 2:**
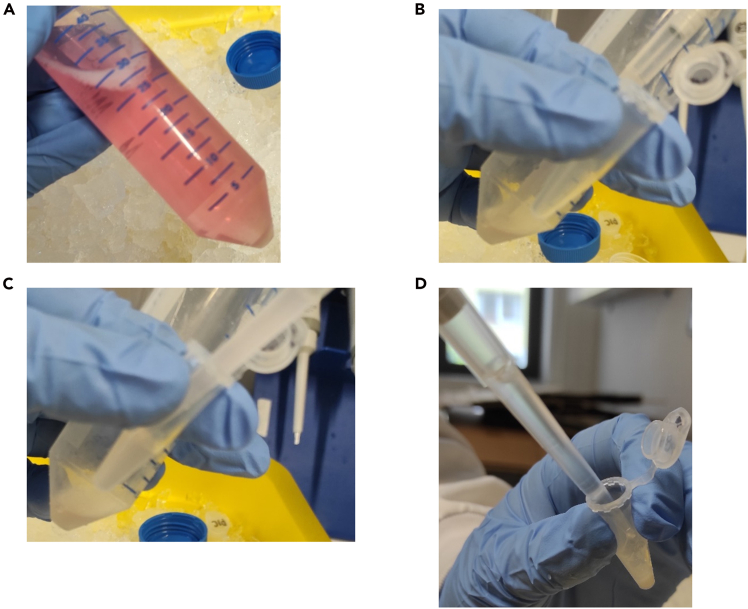
How to wash and lyse the cells (A) Visible cell pellet after centrifuging the cells. Ensure that you see the pellet clearly. (B and C) Transfer the cells with PBS into a 1.5 mL tube. Ensure that you really transfer all of the cells. (D) Lyse the cells with 1 mL lysis buffer including 4% Protease Inhibitor Cocktail (PIC). Resuspend carefully until no clumps are visible. Place the tube on ice for 30 min.
  b. Centrifuge the cells at 500 × *g*, 4°C and for 5 min.
  c. Discard the supernatant.
  d. Add 1 mL ice cold PBS to the pellet.
    i. Resuspending is not necessary.
  e. Centrifuge the cells at 500 × *g*, 4°C and for 5 min.
  f. Discard the supernatant.

***Note:*** Ensure that the medium is thoroughly removed by washing the cells with PBS.

3. Centrifuge the cells at 500 × *g*, 4°C and for 5 min.
  a. Discard the supernatant.
4. Resuspend the pellet in 1 mL ice cold PBS.
  a. Transfer the cell suspension to a 1.5 mL tube (Figures 2A–2C).

5. Lyse the cells.
  a. Add 4% PIC to the lysis buffer.
  b. Add 1 mL lysis buffer (with 4% PIC) to the cell pellet (Figure 2D).
    i. Carefully resuspend the cells in lysis buffer, ensuring no clumps remain.
  c. Place it on ice for 30 min.
6. Centrifuge the cells.
  a. Centrifuge at maximum × *g*, 15 min, at 4°C.
    i. We are using 17 000 × *g*.
  b. Transfer the supernatant to a fresh 1.5 mL tube.

***Note:*** To ensure more protein concentration it is possible to resuspend every 10 min during the lysis.

**CRITICAL:** Do not touch the pellet. If uncertain, re-centrifuge the sample for 5 min at the maximum speed (× g) at 4°C.

### DISC-IP

**Timing: overnight**

In this step, the DISC is immunoprecipitated from the total cellular lysates to isolate its core components including caspase-8. An aliquot of the lysate is taken prior to immunoprecipitation (IP) to serve as the input control.^20^

**Figure 3:**
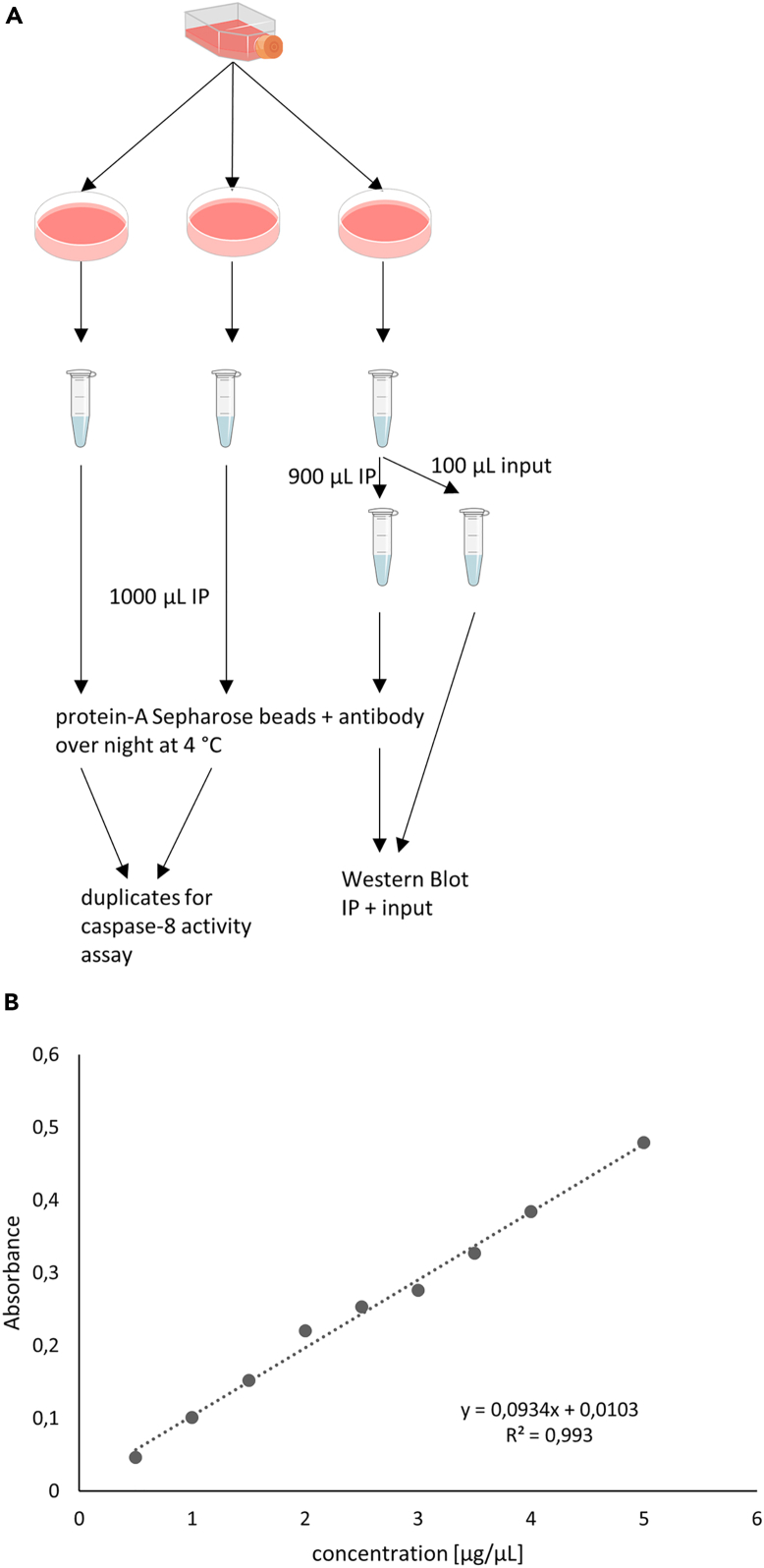
Scheme of the method (A) Cells are seeded from a flask into three 14.5 cm plates per one condition. After the cells are harvested and washed, two samples are directly used for IPs. The third sample is separated in 100 μL for input control and 900 μL are used for IP. IPs from 1000 μL lysate are used for caspase-8 activity assay and IPs from 900 μL lysate are used for Western Blot control together with the input control. (B) An example of the Bradford Standard curve with measured values from the standard protein BSA. A linear regression was fit through the mean of triplicates. Calculating the protein concentration from this curve is only possible in the range between 0.5 and 5 μg/μL. The formula for calculating the protein concentration is shown in the corner as well as the coefficient of determination. Abbreviations: IP Immunoprecipitation.

***Note:*** This sample serves as the expression control for the core DISC components in the western blot analysis.

7. Separating the lysates for DISC-IP and input controls (Figure 3A).
  a. Transfer 100 μL of the lysate into a fresh 1.5 mL tube as input control.
    i. Do this for one lysate per triplicate.

8. Adding the antibodies for IP.
  a. Add 2 μg APO-1 antibodies to each lysate sample to immunoprecipitate the DISC. Perform this for all triplicate samples.
  b. Add 10 μL of protein A Sepharose Beads to each lysate sample for immunoprecipitation. Perform this for all triplicates.
  c. For the ‘Beads control’ add protein A Sepharose Beads only—do not add any antibodies.
  d. Rotate the samples vertically overnight at 4°C.

***Note:*** Ensure your pipette tip can comfortably handle protein A Sepharose Beads. To facilitate this, you may cut the tip to create a wider opening. We recommend using tips with a wide orifice for easier handling.

### Measurement of protein concentration

**Timing: 30 min**

In this step, the protein concentration of the lysates is measured to ensure equal amounts of protein are loaded onto the gel. Additionally this step also serves as a control if the cell lysis was successful and if there is enough protein or if more cells are needed to be seeded. We use the Bradford method for protein quantification.^25^

9. Prepare a standard curve with Bradford.
  a. Mix 10 mg BSA and 10 mL H_2_O.
  b. Prepare a dilution row.
    i. We recommend: 5 mg/mL, 4 mg/mL, 3.5 mg/mL, 3 mg/mL, 2.5 mg/mL, 2 mg/ml, 1.5 mg/mL, 1 mg/mL, 0.5 mg/mL, 0 mg/mL.
    ii. Do it in triplicates.
  c. Fill cuvettes for photometer with 1 ml Bradford substrate.
  d. Add 2 μL of different dilutions to the cuvettes in triplicates.
    i. Use a blank with 2 μL water added to the Bradford substrate.
  e. Mix well by vortexing.
  f. Incubate for 5 min at RT.
  g. Mix again by vortexing.
  h. Measure with a photometer.
    i. Wavelength 595 nm.
    ii. Fit a linear regression between the mean of the triplicates of the values (Figure 3B).

***Note:*** It is only possible to measure in the range of the standard curve.

***Note:*** The standard curve in Figure 3 b is only an example. Prepare your own standard curve.

10. Calculation of the protein concentration.
  a. Fill cuvettes with 1 mL Bradford substrate.
  b. Add 2 μL of the lysates.
    i. Use a blank with 2 μL lysis buffer added to the Bradford substrate.
  c. Mix well by vortexing.
  d. Incubate for 5 min at RT.
  e. Mix again by vortexing.
  f. Measure with a photometer at a wavelength of 595 nm.
  g. Calculate the protein concentration with the standard curve (troubleshooting problem 1) (Figure 3B).

***Note:*** Ensure that the brown Bradford reagent does not turn blue in the absence of protein.

***Note:*** It can be necessary to dilute the lysate with lysis buffer if the protein concentration is too high.

11. Preparation of protein sample for Western Blot.
  a. Take 25 μg protein and prepare a sample of total volume of 15 μL with lysis buffer.
  b. Add Lämmli Sample Buffer.
  c. Incubate the lysates with Lämmli buffer for 5 min at 95°C.
  d. Centrifuge the lysates with Lämmli buffer at 500 × *g*, 1 min, at RT.

***Note:*** You can freeze the lysate at −20°C and do the protein measurement later. Let them thaw on ice.

***Note:*** It is possible to store the lysates with the Lämmli buffer at −20°C for at least 1 year.

***Note:*** Other protein concentration measurements can also be used (for example, the Lowry or Biuret method).

### IP washing and western blot preparation

**Timing: 30 min**

In this step, the protein A Sepharose Beads are washed after overnight incubation with antibodies to remove unbound proteins and reduce nonspecific signals. This process ensures that only target proteins remain bound to the antibodies. The following instructions describe how to prepare these samples for Western blot analysis.

12. Washing IPs.
  a. Centrifuge the cell lysates with the protein A Sepharose Beads at 4°C, 5 min, at 500 × *g*.
  b. Discard the supernatant.
  c. Add 1 mL ice cold PBS to the protein A Sepharose Beads.
    i. Resuspending the cells is not necessary.
  d. Centrifuge at 4°C, 5 min, at 500 × *g*.
  e. Discard the supernatant.
  f. Repeat this step three more times.

**CRITICAL:** Be careful to not touch the pellet as it is hard to see.

13. Dry the protein A Sepharose Beads with a Hamilton pipette.
  a. Discard all the liquid from the protein-A Sepharose Beads until they are dry and look like a white powder.

***Note:*** The Hamilton pipette has a very fine needle where the protein A Sepharose Beads are not fitting. This makes the Hamilton perfect for drying the protein A Sepharose Beads.

14. Preparation of Western Blot control. One of the triplicates are for Western Blot input control (the one where the lysate control was taken from) (Figure 3A).
  a. Add 20 μL Lämmli buffer to the protein A Sepharose Beads of the Western Blot control.
  b. Incubate the protein A Sepharose Beads with Lämmli buffer for 10 min at 95°C.
  c. Centrifuge the protein A Sepharose Beads with Lämmli buffer at 500 × *g*, 1 min, at RT.

***Note:*** It is possible to freeze and store the protein A Sepharose Beads after this step at −20°C for at least 1 year.

### Caspase-8 activity assay

**Timing: 7 h**

In this step, the caspase-8 activity is measured at the DISC, which was immunoprecipitated using protein A Sepharose Beads in step 13.

15. Preparation of samples for caspase-8 activity measurement.
  a. Resuspend the protein A Sepharose Beads from the other two triplicates which are not for Western Blot (Figure 3A) in 95 μL DTT buffer.
  b. Transfer the protein A Sepharose Beads in DTT buffer in a 96-well plate.

***Note:*** Use a cut tip or a tip with wide opening to transfer the protein A Sepharose Beads.

16. Add 100 μL Caspase-Glo 8 substrate (Promega, Germany) to the 96 Well plate.
  a. Add to two wells 100 μL DTT buffer without protein A Sepharose Beads and 100 μL Caspase-Glo 8 substrate as blank control.
  b. Incubate plate for 1 h at RT in the dark.
  c. Measure the luminescence in an appropriate device (troubleshooting problem 2).
    i. We used the TECAN infinite M200 PRO.
    ii. Measurement is in accordance to manufacturer’s instructions.
    iii. https://www.promega.de/products/cell-health-assays/apoptosis-assays/caspase_glo-8-assay-systems/?catNum=G8200.
    iv. The luminescence was measured at the various time points indicated in the table.

**Table:** 

Step	Time	Repetitions
1	5 min	5×
2	10 min	4×
3	30 min	10×

***Note:*** The different incubation times and repetitions allow for an overview of caspase-8 activation dynamics across various time points.

### SDS PAGE and western blot

**Timing: 1 h**

In this step, both the immunoprecipitation (IP) samples and input controls are loaded onto a gel to verify the efficiency of the DISC-IP. The proteins are then transferred onto a membrane for analysis by Western blot using specific antibodies.

17. Prepare a gel as described in the materials and equipment setup chapter.
  a. Load the gel with the denaturated lysates and IP samples.
  b. Load a protein ladder next to the samples.
  c. Let the gel run at 80 V.
  d. Stop the gel when the running front reaches the bottom of the gel.

***Note:*** To save time it is possible to run the gel after 30 min at 80 V with 120 V.

18. Prepare two filter stacks and one membrane for Western Blot by soaking it in Blotting Buffer (Bio-Rad, Germany) for at least 10 min.
  a. Put one filter stack in the blotting cassette.
    i. Carefully remove any air bubbles by gently rolling a blot roller over the filter stack.
  b. Place membrane on the filter stack.
    i. Carefully remove any air bubbles by gently rolling a blot roller over the membrane.
  c. Place gel on the membrane.
    i. Carefully remove any air bubbles by gently rolling a blot roller over the gel.
  d. Place the second filter stack on the gel.
    i. Carefully remove any air bubbles by gently rolling a blot roller over the filter stack.
  e. Close the cassette and put it in the blotting device (Bio-Rad, Germany).
    i. Blot the proteins on the membrane for 12 min with 2.5 A and 25 V.

***Note:*** We are using the Transblot Turbo Kit from Bio-Rad (Germany) for blotting, but other established options are also possible.

19. Place the blotted membrane in 5% milk prepared in PBST to block nonspecific binding sites.
  a. Shake the membrane horizontally with 38 rpm for 1 h at room temperature.

### Western blot detection

**Timing: overnight incubation, 2 h of detection**

In this step, the membrane is incubated with primary and secondary antibodies and the detection of the Western Blot signals is described.

20. Wash the blocked membrane three times for 5 min with PBST.
  a. Add primary antibodies to the membrane.
    i. Use the concentration of antibody diluted in PBST as depicted in the key resources table.
    ii. Add 1:100 sodium azide (NaN_3_) to a final dilution of 1:100 from a 1.5 M stock solution to preserve the primary antibody solution for storage.
  b. Shake the membrane with the primary antibodies horizontally with 38 rpm overnight at 4°C.

***Note:*** We recommend starting with the less efficient antibody for detection.

***Note:*** It is also possible to incubate the membrane with the primary antibodies for 2 h at RT. However, this is only recommended for highly potent primary antibodies.

21. Discard the primary antibody (it can be used several times depending on the antibody).
  a. Wash the membrane three times for 5 min with PBST.
22. Add 20 mL 5% milk in PBST and 1:10000 corresponding secondary antibody.
  a. Incubate the membrane with gentle horizontal shaking at 38 rpm for 1 h at RT.
23. Wash the membrane three times for 5 min with PBST.
  a. Add HRP substrate to the membrane and proceed immediately with the next step.
24. Detect the signals using an imager (troubleshooting problems 3 and 4).
  a. We are using the Chemoluminescence Imager (Bio-Rad, Germany).
  b. Wash the membrane three times for 5 min with PBST.
25. Add the next primary antibodies and start again with 20 a.

***Note:*** We recommend the following antibodies to check DISC formation and extrinsic apoptosis induction: c-FLIP, procaspase-10, procaspase-8, FADD, RIPK1, CD95, procaspase-3, PARP1, actin (or another loading control) (key resources table).

***Note:*** The antibodies should also detect the cleaved forms of the proteins.

***Note:*** The analysis is performed without stripping the membrane, using a sequential antibody incubation strategy based on different IgG isotypes. Typically, the process begins with IgG1 antibodies, followed by IgG2b, and concludes with polyclonal antibodies. This stepwise approach allows for multiple targets to be probed on the same membrane without cross-reactivity.

## Expected outcomes

Caspase-8 activation at the DISC is a key event in the induction of apoptosis**.** This protocol provides a detailed description of how to measure caspase-8 activity at the DISC.

It yields two distinct types of results that together allow conclusions about caspase-8 activation: a caspase-8 activity assay and Western Blot analysis of the DISC immunoprecipitation (DISC-IP).

**Figure 4:**
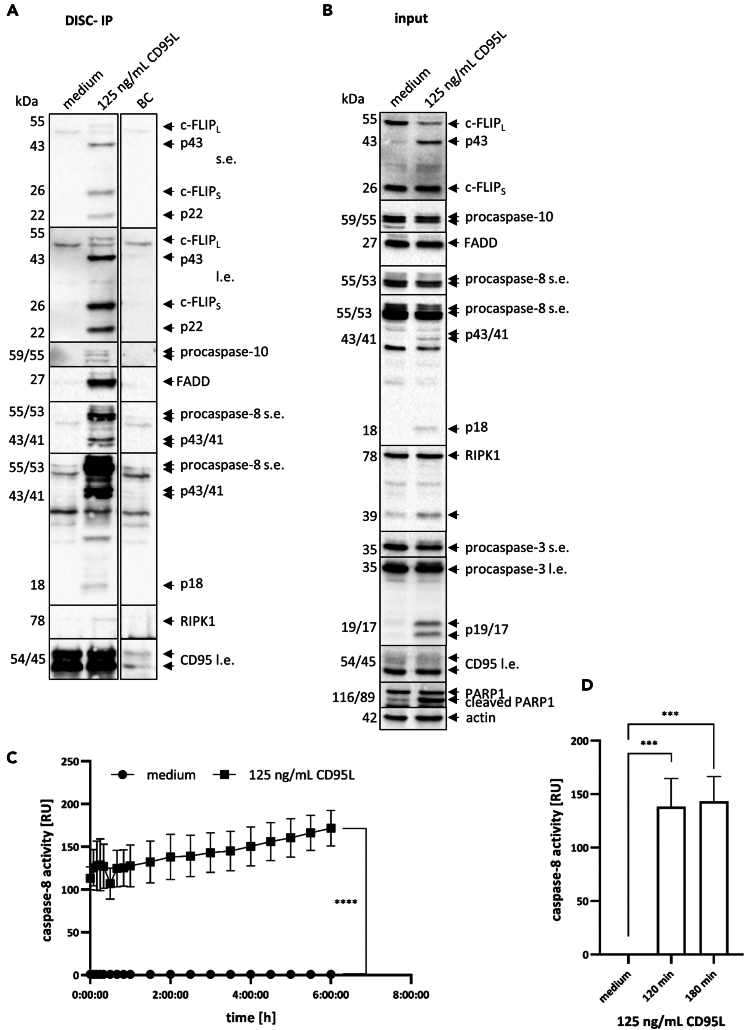
CD95L induced caspase-8 activity at the DISC as measured by caspase activity assays (A and B) HeLa-CD95 cells were treated with CD95L for 2 h. Afterwards cells were harvested and DISC-IP was carried out using anti-APO1 antibodies. DISC-IP was analyzed using Western Blot with the corresponding antibodies. CD95 served as loading control for the IP (A) and actin served as loading control for the input (B). One representative Western Blot out of three is shown. (C and D) HeLa-CD95 cells were treated with CD95L for 2 h. Afterwards cells were harvested and DISC-IP was carried out using anti-APO1 antibodies. Caspase-8 activity at the DISC was measured using the Caspase-Glo 8 assay (Promega). All values are shown over the time (C) or two time points (120 and 180 min) were selected to show the bar diagram (D). Mean and standard deviation from three independent experiments are shown. Statistical analyzes were done with unpaired One-way ANOVA test with Tukey post hoc test. The following values were used: ∗∗∗∗*p* < 0.0001,∗∗∗*p* < 0,001; ∗∗*p* < 0,01; ∗*p* < 0,05; ns not significant Abbreviations: s.e. short exposure, l.e. long exposure, IP Immunoprecipitation, BC Beads control.

Binding of a DL to its DR leads to the formation of the DISC including FADD, procaspase-8/10, c-FLIP as well as cleaved and activated forms of caspase-8/10 and c-FLIP. The DISC-IP should show these proteins and their cleaved and active forms in the Western Blot (Figures 4A and 4B). The input control of the DISC-IP should show all core components of the DISC, including their cleavage products. Additionally, Western blot analysis of the input allows for the detection of further substrates of active caspase-8 and other apoptotic markers, such as cleaved caspase-3. Caspase-3-mediated PARP1 cleavage, a hallmark of apoptosis, can also be detected, with the cleaved form of PARP1 expected to appear following DL treatment. For the DISC-IP, CD95 serves as a loading control, and its signal should be consistent across all conditions. Similarly, a lysate loading control such as actin should show comparable expression levels between samples.

The caspase-8 activity assay should show almost no increase of caspase-8 activity in untreated cells. However the treatment of the cells with DL should result in an induction of caspase-8 activity (Figures 4C and 4D). This caspase-8 activity should increase over time as the caspase-8 activity is measured over longer time intervals. A difference between untreated and treated samples should be clearly visible. The introduction of caspase-8 mutants should lead to the decrease in caspase-8 activity as described in.^1^ The duplicates should not differ too much from each other. It is possible to show all values with a line diagram or to select values and show a bar diagram (Figures 4C and 4D).

## Quantification and statistical analysis

To analyze the DISC-IP and input signals by Western blot, merge the membrane images obtained after antibody detection with the image of the corresponding protein ladder. We use Image Lab software for this purpose, but any other suitable imaging software can also be used. Including the protein ladder alongside the detected protein bands allows for accurate identification of band sizes. Be sure to highlight the detected protein bands, label them with the corresponding protein names, and indicate the molecular weights of the detected bands.

For caspase-8 activity assays, calculate the data as follows: For each time point, begin by calculating the mean luminescence value of the blank samples measured in duplicate. Subtract this mean from all other luminescence values at the same time point to correct for background (unspecific) luminescence. After blank correction, calculate the mean luminescence value of the medium control samples, also measured in duplicate, independently for each time point. Then, normalize all blank-corrected luminescence values by dividing each by the corresponding mean of the medium control at the same time point. This results in relative caspase-8 activity values normalized to the medium control for each time point. To normalize the luminescence values, divide each luminescence measurement by the mean luminescence value of the corresponding medium control at the same time point. Perform this normalization separately for each time point. For example, normalize the values at the 5-min time point by dividing them by the mean luminescence value of the medium control at 5 min. Repeat this procedure for all other time points accordingly.

Calculate the mean and standard deviation of the normalized caspase-8 activity values for each time point. Plot the data over time as a line graph with error bars representing the standard deviation. Alternatively, you may choose a single time point to display in a bar chart with standard deviation (see Figures 4C and 4D). Refer to troubleshooting problem 5 for additional guidance.

We recommend repeating the experiment at least three times. To summarize the overall results, calculate the mean and standard deviation from the means obtained in each independent experiment. Plot the aggregated data over time as a line graph with error bars representing the standard deviation. Alternatively, a single time point can be displayed in a bar chart with standard deviation (see Figures 4C and 4D).

For statistical analyses we recommend an unpaired ANOVA test with a post hoc Tukey test to include all the data in the statistical analyses. The following values should be used: ∗∗∗∗*p* < 0.0001,∗∗∗*p* < 0.001; ∗∗*p* < 0.01; ∗*p* <0.05; ns not significant.

## Limitations

This protocol has not been tested with other immunoprecipitations (IPs), such as FADD- or c-FLIP-IP. While we are confident it could work with these IPs, it is important to note that they are not specific to the DISC and may result in co-immunoprecipitation of other caspase-8-containing complexes such as complex II.^26^ These include the ripoptosome, which contains RIPK1, c-FLIP, and FADD and can lead to apoptosis, as well as the FADDosome, which is involved in NF-κB activation.^27^^,^^28^^,^^29^

There is also a problem in repeating the experiment three times as the luminescence values might be very different between the repetitions, which leads to high standard deviations, which could result in difficulties calculating the significance.

Choosing the right time point can be difficult and requires some preliminary experiments.

## Troubleshooting

### Problem 1

Low protein concentration (related to step 10g).

### Potential solution

• Not enough cells seeded before stimulation. Ensure that you have enough cells in the plate before you stimulate. Check that you do not have less than 70% confluent cells.
• If you have cells that do not grow densely, use more than one plate and mix the cell suspension before lysing the cells.
• Ensure that you scraped all the cells from the plate.
• Ensure that you see a clear pellet after each centrifugation step and that you have completely transferred the pellet after the first centrifugation step.
• Ensure that you never touched the pellet when you discarded the supernatant.
• Ensure that you carefully resuspended the cell suspension into the lysis buffer.
• Ensure that your cells are still attached to the surface when you discarded the old media and added the fresh media. It is possible that you have to carefully change the media as the cell can become detached due to the pipetting (Figures 1A and 1B).

### Problem 2

No or only small increase in caspase-8 activity (related to step 16c).

### Potential solution

• This could be a sign of wrong or non-efficient treatment. Run calibration experiments before starting with this protocol. Calibration experiments could be a concentration row with the compound you want to use for stimulation. These experiments can be performed in the time- and dose-dependent manner. You could do calibration experiments with measuring the caspase-8 activity without the DISC-IP. Then you get all caspase-8 activity in the cell and not only at the DISC but this could give you a hint on the best time interval and concentration. It is also possible to do time- and dose- dependent calibration experiments and detect the caspase-8 processing with Western Blot and use this information to determine the best conditions for experiments.
• This could be also a sign of the wrong time point. Make preliminary experiments with different time points as described in the point above.

### Problem 3

Weak or bad signals in Western Blot (related to step 24).

### Potential solution

• This could be due to little amount of antigen present in the sample, due to the low amount of protein after cell lysis (problem 1) or low activity of the primary antibodies.
• You can increase a concentration of primary antibodies used.
• Ensure that when the membrane is blocked or when you add primary or secondary antibodies the membrane is covered completely with the corresponding solution.
• Ensure that you wash the membrane at least three times with PBST before addition of the secondary antibodies.
• Ensure that the exposure time is sufficient to detect the antibody signal clearly.
• Use the antibodies in accordance to manufacturer’s instructions.

### Problem 4

No DISC formation in the Western Blot visible (related to step 24).

### Potential solution

• This could be due to little amount of protein after cell lysis (problem 1).
• The timing or concentration of the used compound could be wrong (problem 2).
• This could be due to the low efficiency of antibodies (problem 3).
• Ensure that you had protein A Sepharose Beads in the lysates. It can be hard to add protein A Sepharose Beads to the lysate as they do not fit in a normal tip.

### Problem 5

Big differences between repetitions leading to high variances (related to step Quantification).

### Potential solution

• Make sure that every step is done in a same way in the repetitions.
• Make sure that the cells have a similar viability before seeding them in the plates. Use only cells which have a viability higher than 93%.
• Ensure that you have similar amount of cells in the plates by checking it with a microscope.
• Ensure that you had similar amounts of protein A Sepharose Beads added to the lysate (problem 4).

## Resource availability

### Lead contact

Further information and requests for resources and reagents should be directed to and will be fulfilled by the lead contact, Inna N. Lavrik (inna.lavrik@med.ovgu.de).

### Technical contact

Technical questions should be directed to and will be fulfilled by the technical contact, Corinna König (corinna.koenig@ovgu.de).

### Materials availability

Reagents generated in this study will be made available on request, but we may require a completed materials transfer agreement.

### Data and code availability

Data generated in this study will be made available on request.

## Acknowledgments

We acknowledge the project ALBB (European Regional Development Fund), InMedAK (European Regional Development Fund), and 10.13039/501100001659DFG (LA 2386).

## Author contributions

Manuscript writing and experiments, C.K.; manuscript writing and supervision, I.N.L.

## Declaration of interests

The authors declare no competing interests.
